# Development of Graphene/Recycled Carbon Fiber-Reinforced PLA Composites for MEX Printing and Dry Machinability Analysis

**DOI:** 10.3390/polym17172372

**Published:** 2025-08-31

**Authors:** Abdullah Yahia AlFaify, Mustafa Saleh, Saqib Anwar, Abdulrahman M. Al-Ahmari, Abd Elaty E. AbdElgawad

**Affiliations:** Industrial Engineering Department, College of Engineering, King Saud University, P.O. Box 800, Riyadh 11421, Saudi Arabia; msaleh3@ksu.edu.sa (M.S.); sanwar@ksu.edu.sa (S.A.); alahmari@ksu.edu.sa (A.M.A.-A.); aesayed@ksu.edu.sa (A.E.E.A.)

**Keywords:** 3D printing, PLA, graphene reinforced polymer, recycled carbon fibers, carbon fibers reinforced polymer, MEX, ball milling, 3D-printed polymers machining

## Abstract

Material extrusion (MEX) is an additive manufacturing process used for 3D printing thermoplastic-based polymers, including single polymers, blends, and reinforced polymer composites (RPCs). RPCs are highly valued in various industries for their exceptional properties. The surface finish of RPC MEX-printed parts is high due to the process-related layering nature and the materials’ properties. This study explores RPC development for MEX printing and the potential of dry milling post-processing to enhance the MEX-printed part’s surface quality. RPC MEX filaments were developed by incorporating graphene nanoplatelets (GNPs) and/or recycled-carbon fibers (rCFs) into a polylactic acid (PLA) matrix. The filaments, including pure PLA and various GNPs-PLA composites, rCF-PLA, and rCF-GNPs-PLA, were developed through ball mill mixing and melt extrusion. Tensile tests were performed to assess the mechanical properties of the developed materials. Dry milling post-processing was carried out to assess the machinability, with the aim of enhancing the MEX-printed part’s surface quality. The results revealed that adding GNPs into PLA showed no considerable enhancements in the tensile properties of the fabricated RPCs, which is contrary to several existing studies. Dry milling showed an enhanced surface quality of MEX-printed parts in terms of surface roughness (Sa and Sz) and the absence of defects such as delamination and layer lines. Adding GNPs into PLA facilitated the dry machining of PLA, resulting in reduced surface asperities compared to pure PLA. Also, there was no observation of pulled-out, realigned, or naked rCFs, which indicates good machinability. Adding GNPs also suppressed the formation of voids around the rCFs during the dry milling. This study provides insights into machining 3D-printed polymer composites to enhance their surface quality.

## 1. Introduction

Additive manufacturing (AM), commonly known as 3D printing, stands out as a technological marvel that enables the production of complex geometrical products with high precision in reduced timeframes [1]. Various variants of AM technologies are invented with a specific set of dimensional, aesthetic, and structural features. Material extrusion (MEX) is a polymer-based AM process and is the most commonly used AM process, where thermoplastic materials in the form of filaments are melted and deposited layer-by-layer to fabricate a part [2]. MEX is capable of processing a wide range of polymer-based materials, including reinforced polymer composites (RPCs) [3,4,5], single polymers [6], and blends [7]. In general, MEX is known for its simplicity, low cost, availability, and high-quality structural properties [5]. However, MEX-printed parts exhibit process-related surface imperfections [8], e.g., staircase, resulting in unsatisfactory surface finish [2] and diminished mechanical and physical properties [1]. MEX-printed parts typically exhibit rougher surfaces, which could be attributed to many reasons, such as the staircase effect, layer lines, thermal contraction of the material during cooling, layer-to-layer adhesion, layer thickness, and reinforcements. Surface quality deficiencies in the MEX-printed parts are typically addressed through pre-processing and post-processing processes [2,8]. In the pre-processing, MEX printing parameters (e.g., layer thickness and building orientation) are optimized for enhancing the surface quality. However, pre-processing is not sufficient for obtaining the parts for applications where relatively high-quality surfaces are required. In this regard, post-processing, e.g., machining, can be an effective method for obtaining high-quality surfaces.

Polymers typically demonstrate lower mechanical, thermal, and electrical properties compared to other materials such as metals. These properties can be enhanced by incorporating reinforcements into the polymer matrix to form RPCs. In RPCs, typical reinforcements include nanoparticles, e.g., graphene nanoplatelets GNPs and aluminum oxide Al_2_O_3_, and fibers, e.g., glass fibers (GFs) and carbon fibers (CFs). In the following, some studies reporting on incorporating GNPs and carbon fibers are reviewed.

Cetiner et al. [9] developed GNP-reinforced polymers, including PLA and polyurethane (TPU), through a melt mixing process. GNPs were incorporated with PLA and TPU with 0.5 wt.%, 1 wt.%, and 2 wt.% loadings. The results showed an enhancement in the flexural strength and thermal conductivity when 0.5 wt.% of GNPs was added to the PLA/TPU blend. However, the tensile strength and modulus were slightly reduced when GNPs were added to the PLA. In [10], ready filaments of PLA/GNPs were used to characterize the influence of adding GNPs into PLA on mechanical properties. The findings showed that adding GNPs enhanced the tensile and flexural strengths, with no influence on the tensile modulus. On the contrary, the addition of GNPs led to a reduction in the impact strength. The influence of the GNP incorporation on the tensile strength was found to be highly dependent on the MEX printing orientation. Vidakis et al. [11] evaluated the addition of GNPs into PLA on the mechanical properties by conducting different mechanical tests, including tensile, flexural, compression, and hardness tests. The findings showed that PLA and PLA/GNP polymers showed similar behavior in the considered mechanical properties, with slight domination of the pure PLA. In [12], researchers incorporated 0.1 wt.% of graphene-based reinforcements, including GNPs, graphene oxide (GO), and nano graphite particles, into wheat straw/PLA material. Mechanically tested samples were produced by the hot-pressing process. The composite materials showed significant enhancement in thermal stability and mechanical properties, including flexural strength, tensile strength, and tensile toughness, in comparison to pristine wheat straw/PLA. Kim et al. [13] studied the influence of reinforcing PLA with different GNP loadings (1 wt.%, 2 wt.%, 3 wt.%, 4 wt.%, and 5 wt.%). Up to 2 wt.%, both tensile strength and elongation at break were significantly enhanced, while tensile modulus was decreased.

Similarly, several studies have attempted to incorporate the CFs in the polymers for MEX-based printing. For instance, incorporating short CFs (SCFs) into polypropylene (PP) for developing MEX materials (PP/SCFs) enhanced the tensile strength by up to 150% and the impact energy by up to 260% in comparison to the neat PP [14]. However, the composites become brittle, leading to a decrease in the break strain. In [15], the development of MEX composites using PP and SCFs (PP/SCFs) enhanced the printed parts’ tensile strength, bending strength, and thermal and electrical conductivity. Additionally, the results demonstrated that printing parameters significantly impact the performance of the developed composite samples. In regard to the significance of CFRPs, producing CFRPs from recycled CFs (rCFs) provides multiple benefits, including decreasing negative impacts on the environment and energy consumption [16] and improving mechanical properties [17,18,19,20].

As mentioned earlier, MEX-printed parts need post-processing to enhance their surface quality. However, very limited research studies have been reported on the machinability analysis of MEX-printed RPCs. Ferreira et al. [21] studied the face milling machinability analysis of MEX-printed polyamide 12 (PA12) and PA12/CF. A significant decrease in surface roughness for both materials was reported after face milling. Moreover, the incorporation of CFs enhanced the machinability, showing less cutting forces and tool wear compared to PA12. Guo et al. in [22] conducted dry milling on MEX 3D-printed PEEK and CF/PEEK to enhance their surface quality. The results demonstrated that dry milling of 3D-printed PEEK and CF/PEEK parts considerably enhances the surface quality. Cococcetta et al. [23] studied the dry and minimum quantity lubrication (MQL) milling postprocessing on 3D-printed CFRP composites. The results showed that post-process machining of 3D-printed CFRP composites enhanced the surface quality finish. The surface finish, burr formation, and tool wear were significantly reduced in MQL machining compared to dry machining. Reference [24] studied the influence of different cooling methods, including dry, MQL, and cryogenic, during milling of 3D-printed Onyx composites. Their findings showed that cryogenic machining considerably improved surface finish and reduced burr formation and tool wear. Reference [25] applied face milling and peripheral milling as post-processing of MEX-printed PETG and CF-PETG. Machinability characteristics, including energy consumption, dimensional accuracy, flatness, and surface roughness, were investigated. The results indicated that incorporating CFs into PETG reduced energy consumption in both operations and improved the surface quality during face milling. Peripheral milling of CF-PETG at higher layer thickness results in significant surface defects, including tearing and burr formation.

It can be seen from the reviewed literature that machining post-processing is helpful in enhancing the surface quality of the MEX-printed parts. Despite that, machining polymer-based composites (e.g., MEX-printed CFRPs) is regarded as a challenging task due to their anisotropic and heterogeneous structure, which can lead to delamination, splintering, and fractures [25,26]. Moreover, machining CFRPs can lead to surface quality problems resulting from fiber breakage and delamination. One of the reasons for the aforementioned machining non-conformities (fiber breakage and delamination) is the lack of lubrication. Due to the hygroscopic properties of polymer-based materials (e.g., CFRPs), which can cause moisture-related issues such as swelling, adsorption, hydrolysis, and changes to the mechanical properties [27], liquid-based lubrication (e.g., emulsions and MQLs) may not be ideal for machining such materials. The inclusion of the GNPs within the CFRPs can possibly enhance their machinability due to the lubricative nature of GNPs.

To the best of our knowledge, there is no comprehensive exploration of developing rCF- and GNP-reinforced polymers for machining investigation. The goal of the current work is to evaluate the potential of reinforcing PLA and CFRP (PLA/rCF) composites with GNPs for 3D printing and then perform a detailed machining analysis of these composites to investigate the effect of GNPs on the machined surface quality. For this purpose, different percentages of GNPs (0.125 wt.%, 0.5 wt.%, and 1 wt.%) were incorporated into the PLA matrix to develop MEX composite filaments. PLA composite filaments with 5 wt.% of rCFs were also developed. Furthermore, GNP-coated rCFs were incorporated with PLA to produce PLA/rCF/GNP composites having 5 wt.% rCFs and 0.5 wt.% GNPs. The mechanical properties of the developed composites were evaluated, and then post-processing by dry milling operation was conducted to explore the machinability of these composites.

## 2. Materials and Methods

### 2.1. Materials

Polylactic acid (PLA) biopolymer (Ingeo 4043D, Natureworks) was obtained from 3DXTech (Grand Rapids, MI, USA). The PLA was supplied in the form of pellets. The GNPs and desized recycled carbon fibers (rCFs) were used as reinforcements. GNP (NG01GNP0109) reinforcement was obtained from Nanografi (Jena, Germany). The rCFs (CarbisoTM MLD/MF-100) were obtained from Procotex (Dottignies, Belgium). The properties of PLA, GNPs, and rCFs, as detailed in the manufacturers’ technical datasheet, are reported in Table 1.

### 2.2. MEX Filaments Production

**Figure 1:**
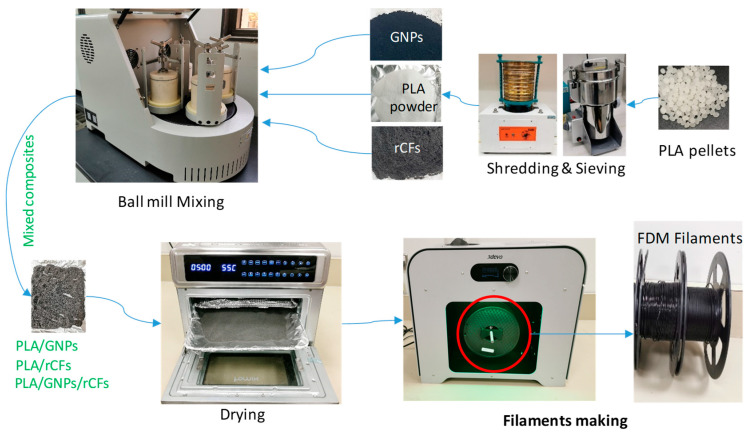
Development of PLA-composite filaments for MEX printing. Reproduced with permission [20].

The process of developing MEX filaments is shown in Figure 1 and described in detail in our previous work [20]. It consists of three main phases: material preparation, mixing, and extruding filaments. The summary of the three phases is as follows.

#### 2.2.1. Material Preparation

Mixing PLA in the form of pellets with micro (rCF) or nano (GNP) reinforcements leads to the reinforcement agglomeration and non-uniform distributions of rCFs and GNPs in the PLA. Thus, the PLA pellets are first ground and converted into powder with a size less than 600 µm before mixing. Converting PLA pellets into powder form involves a two-step process of shredding and sieving (Figure 1), which was used for further mixing.

#### 2.2.2. Mixing

PLA was mixed with varying concentrations of GNPs and rCFs (see Table 2) using a planetary ball mill (TENCAN XQM-2A, Tianchuang Powder Tech, Changsha, China), as depicted in Figure 1. The materials were dried before and after the mixing process. Table 3 shows the employed parameters in the ball mill mixing process.

#### 2.2.3. Filament Making

MEX filaments at various loadings of GNPs and rCFs listed in Table 2 were produced using the extrusion process, see Figure 1. A single screw extruder, called Composer 350 (3DEVO, Utrecht, The Netherlands) equipped with four heating zones was used. The extrusion settings employed to produce filaments with a 1.75 mm average diameter are illustrated in Table 4.

### 2.3. MEX Printing

MEX printing of the developed materials was carried out using a Prusa i3 MK3S+ MEX printer (Prusa Research, Prague, Czech Republic). Various samples, including tensile testing samples, samples for morphological analysis, and samples for machining evaluations, were designed and MEX-printed. The MEX printing parameters used for printing the different samples are illustrated in Table 5.

### 2.4. Morphology Characterization

The morphological characteristics, including filament surfaces, the printed samples’ interlayer and intralayer morphologies, fracture morphology, and machined surfaces, were assessed using a scanning electron microscope (SEM) from JEOL (JCM 6000 plus, Tokyo, Japan).

The ImageJ 1.54g program was used to measure the diameter of the rCFs from SEM images before and after extruding. Samples of the produced composite filaments were heated to 450 °C to evaporate the PLA matrix, and then rCFs were collected and measured.

### 2.5. Tensile Testing

**Figure 2:**
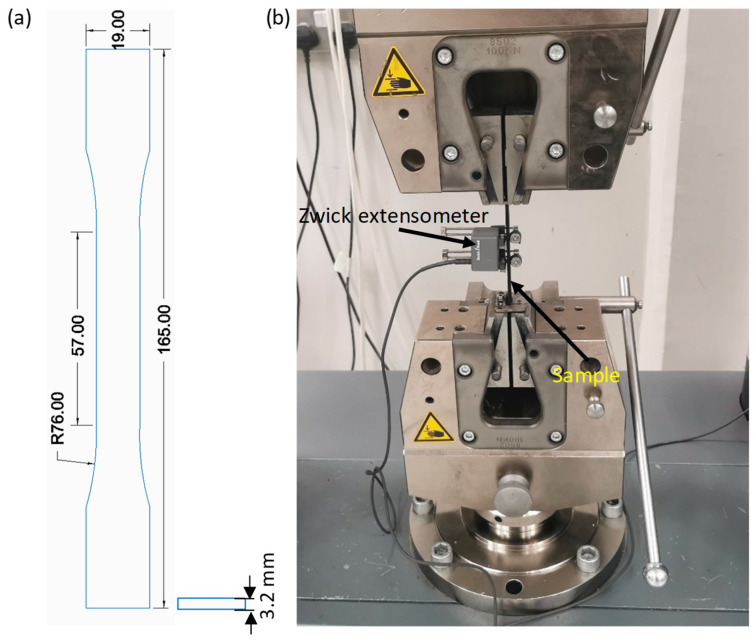
(**a**) tensile testing sample (ASTM D638) and (**b**) tensile testing setup.

Uniaxial tensile testing was performed according to the ASTM D638-14 standard [28] (see Figure 2a) to evaluate different mechanical properties, including tensile modulus (E), yield strength ($Y_{0.2\%}$, i.e., 0.2% offset yield strength), ultimate tensile strength (UTS), and elongation at break (*ε_b_*), of the developed materials. Tests were conducted using a Zwick Roell Z100 UTM (ZwickRoell GmbH & Co. KG, Ulm, Germany) at a constant speed of 5 mm/min until failure. Three samples were tested for each material, and their average were reported. The tensile testing setup is shown in Figure 2b.

### 2.6. Machinability Analysis

**Figure 3:**
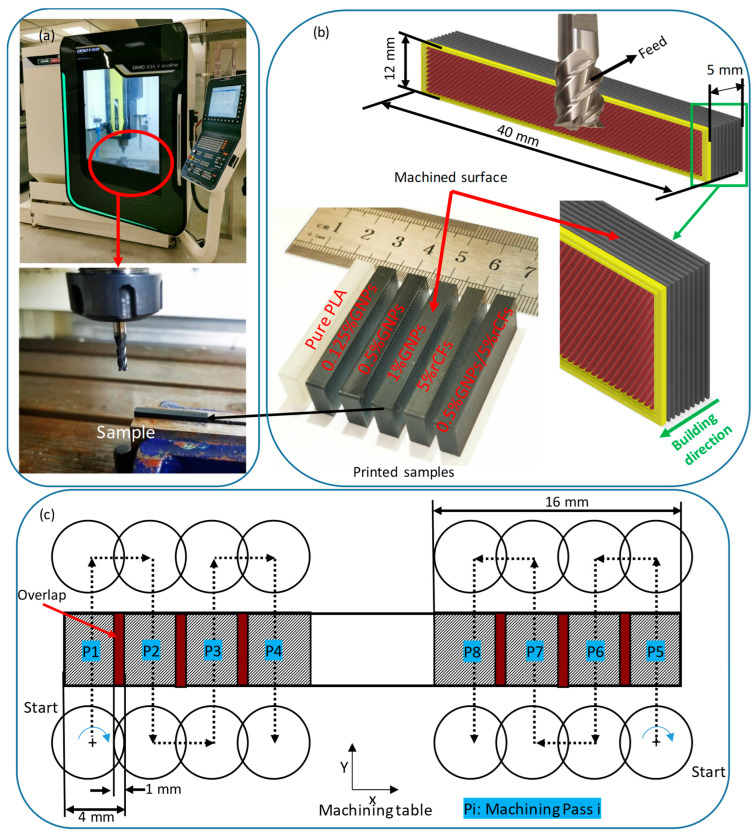
Machining methodology: (**a**) milling machine setup, (**b**) machining samples and machining direction (across layers), and (**c**) schematic diagram of the tool path during face milling machining process.

Post-processing operations were conducted to enhance the surface finish of the MEX-printed samples using a three-axis CNC milling machine (DMC 635 V Ecoline, DMG Mori, Oelde, Germany), Figure 3a. MEX-printed parts typically exhibit rougher surfaces on the surfaces (i.e., side face as shown in Figure 3b) that are parallel to the building direction. This could be attributed to many reasons, such as the staircase effect, layer lines, thermal contraction of the material during cooling, layer-to-layer adhesion, and layer thickness. Thus, the machining process was conducted on the side face that is parallel to the building direction, as shown in Figure 3b.

A sample of 40 mm length, 5 mm width, and 12 mm height of each material listed in Table 2 was printed for the machinability analysis, see Figure 3b. Dry machining process was carried out across the layers, and eight machining passes (represented by “Pi”) were performed on each sample, as shown in Figure 3c. Table 6 shows the employed machining parameters. Milling post-processing operations were conducted at a constant cutting speed, feed rate, and depth of cut, as the aim of this study was to analyze the impact of rCF and/or GNP reinforcements on PLA machinability. The selected machining parameters were based on the literature and preliminary experiments.

The surface roughness of the machined surfaces was evaluated by conducting 3D surface scanning using a 3D optical profilometer (ContourGT-K) from Bruker, Beerlika, MA, USA. An area of 2.279 mm × 1.709 mm was extracted from each 3D scanned surface to obtain the surface roughness, namely Sa. Twelve scans were conducted at each machine repetition, and their average were reported. SEM images were used to study the morphologies of the machined surfaces.

## 3. Results

### 3.1. Morphology Characterization

**Figure 4:**
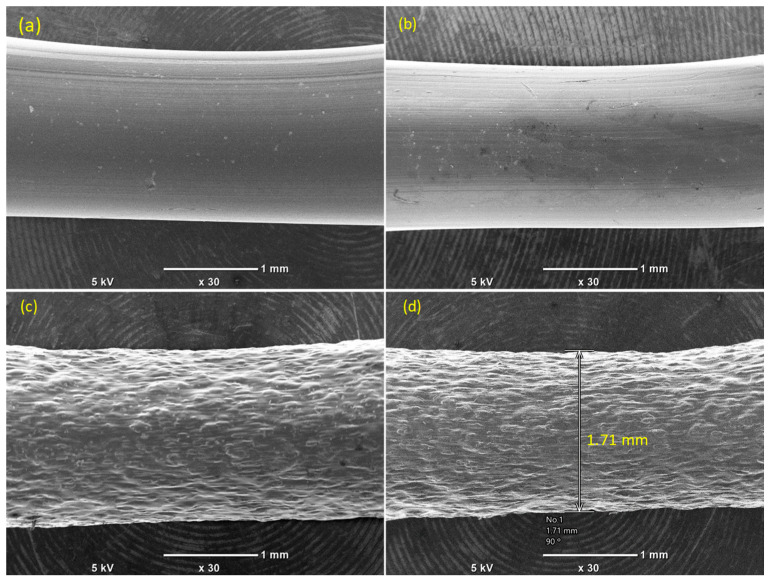
Surface morphologies of the produced filaments: (**a**) PLA, (**b**) PLA/0.5% GNPs, (**c**) PLA/5% rCFs, and (**d**) PLA/0.5% GNPs/5% rCFs.

The surface morphology of the produced filaments was analyzed and depicted in Figure 4. The SEM images demonstrate that filaments composed of pure PLA (Figure 4a) and PLA blended with GNPs (Figure 4b) exhibit smooth surface topographies. However, when rCFs are incorporated into the PLA (Figure 4c,d), the surface becomes rougher in comparison to the pure PLA and PLA/GNP filaments. This is attributed to the presence of rCFs on the extruded filament and the increase in the filament-nozzle friction during the extrusion process. It should be noted that rougher filaments could increase the surface roughness of the MEX-printed parts.

The average length of the as-received rCFs is 89 ± 58 µm, while their average length after extrusion is 85 ± 48 µm, as found in [20]. The average diameter of the as-received rCFs is 8.22 ± 0.93 µm, whereas their diameter post-extrusion is 8.55 ± 0.78 µm. The dimensions of the rCFs (average lengths and diameters) exhibit close agreement before and after extrusion, indicating that rCFs do not undergo changes during mixing and extrusion.

**Figure 5:**
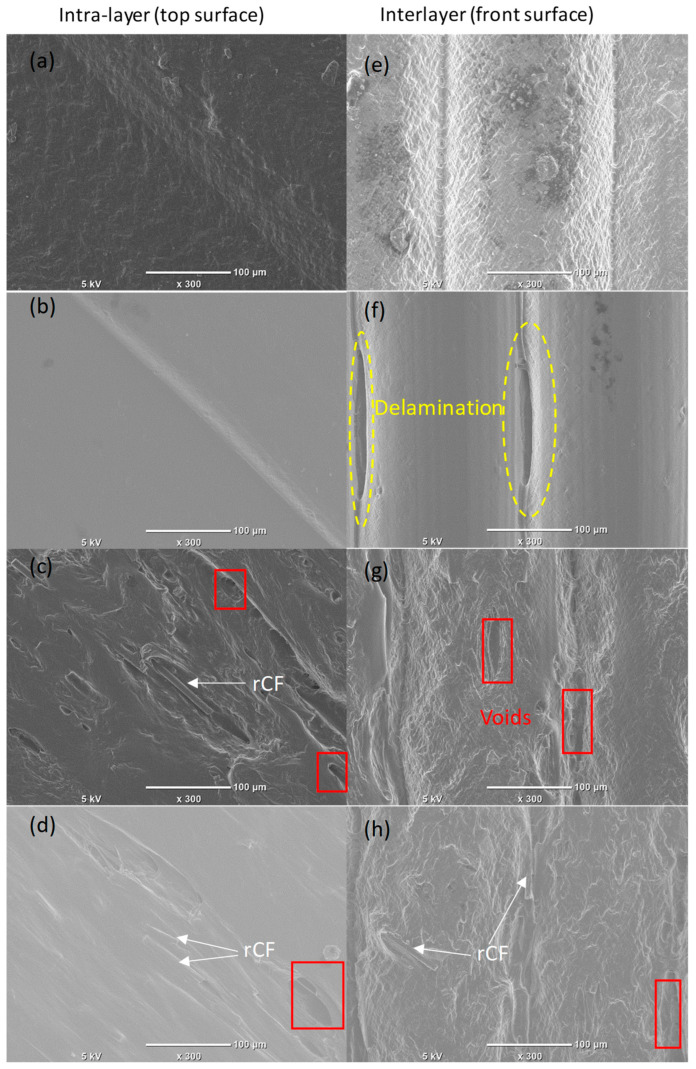
Intralayer and interlayer morphologies of the MEX-printed samples: (**a**,**e**) PLA, (**b**,**f**) PLA/0.5% GNPs, (**c**,**g**) PLA/5% rCFs, and (**d**,**h**) PLA/0.5% GNPs/5% rCFs.

Figure 5 shows the MEX-printed samples’ surface quality, including the intralayer morphology shown in Figure 5a–d and interlayer morphology depicted in Figure 5e–h. The SEM images of the printed samples from both the pure PLA (Figure 5a) and 0.5% GNP (Figure 5b) samples showed high quality in the intralayer morphology, as they showed the absence of defects like voids, cracks, and delamination. Figure 5f exhibited delamination defects between layers (interlayer) for the PLA/0.5% GNP sample on the front side. The samples with rCFs showed voids and naked rCFs on both surfaces. In our previous work [20], we conducted a detailed investigation into the distribution and alignment of rCFs within the PLA matrix. As reported in that study, rCFs were found to be homogeneously distributed throughout the PLA matrix, which highlights the effectiveness of the methodology we employed.

### 3.2. Mechanical Characterization of PLA/rCF/GNP Composites

The mechanical characteristics of the tensile tested samples are summarized in Table 7. The variation in the three repeated experimental results is represented by the standard deviation (SD). In general, low variation among the three repeated samples across the different properties is evident in Table 7, suggesting high repeatability of the proposed methodology for producing and 3D printing the developed composite materials. The weights of the tensile samples demonstrate minimal variation across different materials and repeated samples. This indicates that the produced filaments exhibit consistent properties, including their diameters, resulting in a significantly decreased variation in weights. Significant weight variations caused by filament inconsistency or 3D printing issues, such as clogging, can result in substantial variations in mechanical properties due to either over-extrusion or under-extrusion.

The tensile results showed that the incorporation of GNPs had a slight influence on the tensile modulus. Until GNP loading of 0.5 wt.%, the mechanical properties are similar to those of pure PLA, with slight domination of the PLA/GNP composite, particularly yield strength. However, as the GNP loading increased (1 wt.%), the ultimate strength reduced, and composites became more brittle. These findings are in agreement with [9], in which the strength and elongation at break were reduced as the GNP loading increased from 0.5 wt.% to 1 wt.%. According to [9], this reduction could be attributed to the random alignment of platelet structures through the PLA polymer chains, which resulted in higher stress concentrations. As reported in the literature, a controversial effect of graphene (GNPs) on the mechanical properties has been reported in [9,10,13]. For instance, the addition of GNPs to PLA at 0.5 wt.%, 1 wt.%, and 2 wt.% resulted in a slight decrease in tensile strength and modulus [9]. However, Kim et al. [13] reported that adding GNPs at 1 wt.% to PLLA enhanced both tensile strength and elongation at break, while tensile modulus was decreased. Another work [10] found that adding GNPs to PLA enhanced the tensile strength with no influence on the tensile modulus. These differences could be attributed to many factors, including mixing process, processing method, 3D printing parameters, aging, and filament issues.

**Figure 6:**
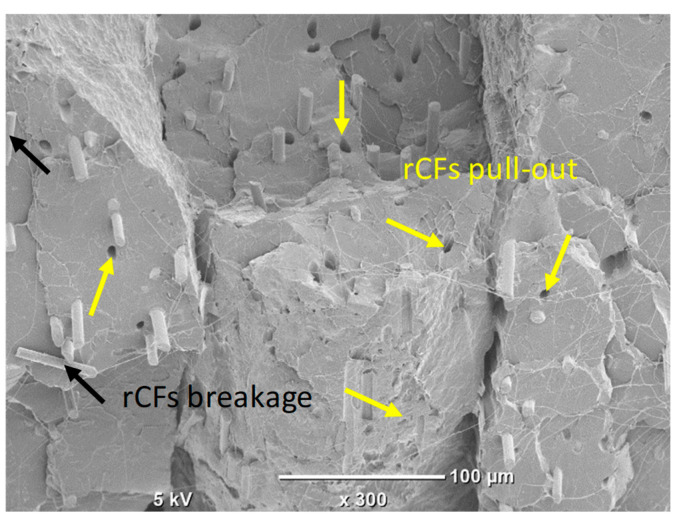
SEM images showing rCFs’ associated defects on a fracture morphology of the tensile-tested PLA/0.5% GNP/5% rCF sample.

Regarding rCFs, the incorporation of 5 wt.% of rCFs increased the tensile modulus by about 15% compared to the pure PLA sample. However, the incorporation of rCFs reduces strength and ductility compared to pure PLA. The decrease in the yield strength and the UTS when adding rCFs into PLA could be attributed to reasons including the presence of voids, rCFs’ breakage and pulling-out (see Figure 5 and Figure 6), and rCFs’ agglomerations.

**Figure 7:**
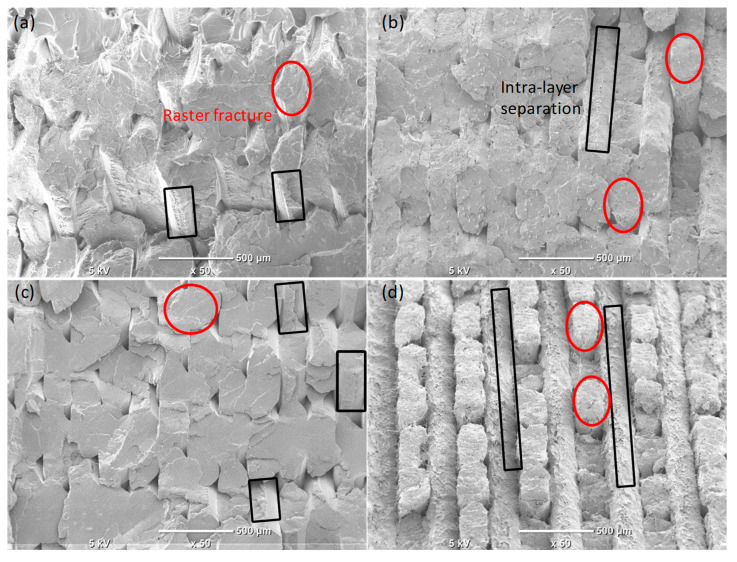
Fractography of the tensile-tested samples: (**a**) PLA, (**b**) PLA/5% rCFs, (**c**) PLA/0.5% GNPs, and (**d**) PLA/0.5% GNPs/5% rCFs.

Figure 7 depicts the fractography of the fractured tensile samples. The dominant failure mechanisms among the different samples are the raster’s fracture (circled in red) and separation between the adjacent rasters, i.e., intralayer separation (elaborated in black rectangles). Intralayer separation is mainly attributed to the raster direction, which in our case is ±45°. The samples with rCF incorporation exhibited rCF pull-out and breakages that contribute to the failure, as illustrated in the high magnification SEM image shown in Figure 6.

### 3.3. Machinability Analysis

**Figure 8:**
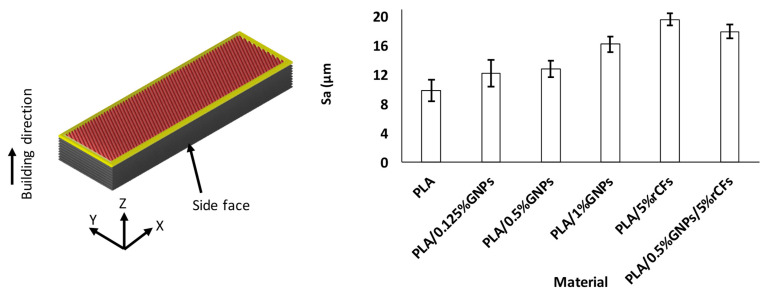
Surface roughness of the as-built samples at the side face.

The surface roughness of the as-built samples’ side face (i.e., a plane that is parallel to the building direction) of the different samples is shown in Figure 8. The findings showed that Sa ranges from 9.9 µm to 19.6 µm and is higher in composite materials, especially in rCF-reinforced PLA, compared to pure PLA, highlighting the need for post-processing to minimize it.

**Figure 9:**
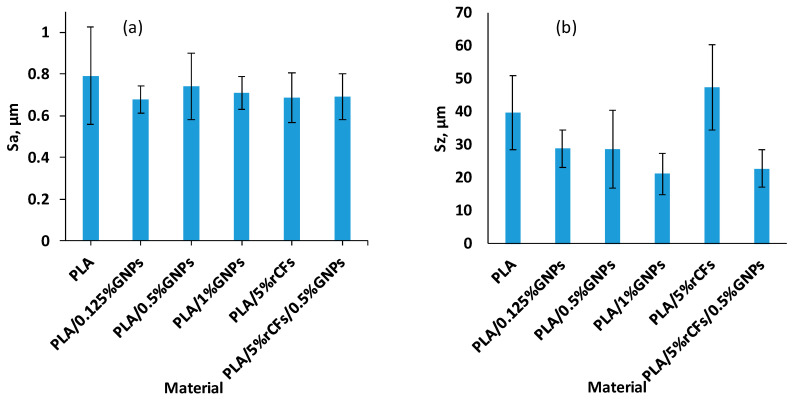
Surface roughness results of the machined surfaces at different GNP and rCF loadings: (**a**) Sa and (**b**) Sz.

Figure 9 shows the surface roughness (Sa and Sz) of the pure PLA and PLA composites after machining across the layers. The Sa results showed no considerable difference between pure PLA and PLA composites, with a slight reduction in roughness for the composite materials. The average Sa was between 0.68 µm at 0.125% GNPs and 0.79 µm for the pure PLA. Figure 9b illustrates the Sz results, emphasizing the substantial improvement in Sz upon the incorporation of GNPs into pure PLA and PLA/5% rCFs. The average Sz for PLA and PLA/5% rCFs are 39.53 µm and 47.32 µm, respectively. However, incorporating 0.5 wt.% GNPs into PLA and PLA/5% rCFs decreases the Sz to 28.57 µm (27.74%) and 22.63 µm (52.17%), respectively. This enhancement, particularly in Sz, could be attributed to the GNPs acting as a lubricant during machining, leading to a smoother surface than neat PLA and PLA/rCF samples. This shows that the Sz parameter is more sensitive in capturing surface asperities while machining polymer composites. This means for precision-machined parts where tolerances are essential, Sz should be considered to evaluate machining performance.

**Figure 10:**
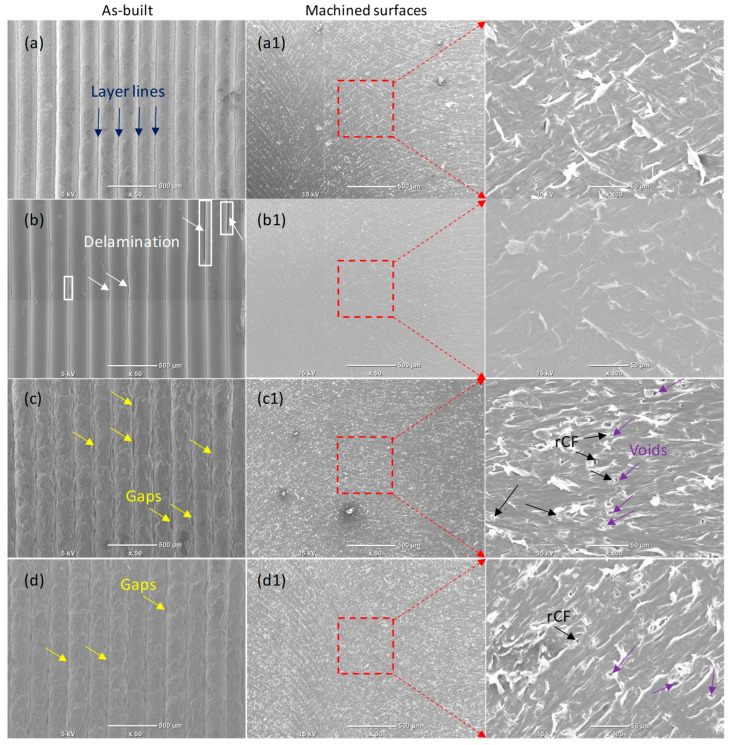
Morphology characteristics of the as-built surfaces (**a**–**d**) and machined surfaces (**a1**–**d1**) across layers: (**a**,**a1**) pure PLA, (**b**,**b1**) PLA/0.5% GNPs, (**c**,**c1**) PLA/5% rCFs, and (**d**,**d1**) PLA/0.5% GNPs/5% rCFs.

Figure 10 presents SEM images of both the as-built and post-machining surface morphologies of MEX-printed samples. The SEM images of the machined surfaces indicate an absence of deposited layer lines, which can be linked to the smearing effect that helps fill the gaps between the layers. Moreover, the absence of cavities and grooves during dry machining suggests that the material removal mainly occurs through a ductile removal mechanism. Both phenomena, polymer smearing and ductile removal, facilitate the removal and leveling of the raised ridges in the deposited layers, thereby decreasing surface roughness and enhancing the quality of MEX-printed polymers, as also highlighted in [22]. The SEM images of the machined surfaces in Figure 10 demonstrate a significant enhancement in surface quality, with a decrease in defects on the machined surfaces compared to the as-built surfaces, which comprise various imperfections, as shown in Figure 5. For instance, the machined surface of the PLA/0.5% GNP sample (Figure 10(b1)) exhibits no interlayer surface defects, such as delamination. In contrast, the as-built sample shown in Figure 5b and Figure 10b for the PLA/0.5% GNP sample shows the presence of such interlayer delamination defects. It should also be noted that defects such as delamination and tearing were not visible for all machined samples, providing further evidence of ductile machining. Significantly fewer surface asperities are noted in the PLA/0.5% GNP sample (Figure 10(b1)) compared to neat PLA (Figure 10(a1)). This could be attributed to the improved lubrication effect and the higher thermal conductivity of PLA/0.5% GNPs, which facilitates higher heat dissipation/extraction from the machined surface, resulting in lesser thermal damage.

The machined surface morphologies of the CFRP samples show that the rCFs are cleanly cut with no observation of pulled-out rCFs, realignments of rCFs, or naked rCFs, which indicates good machinability. The absence of pulled-out rCFs could be attributed to the following reasons: (a) good mechanical interlocking of the rCFs with the PLA matrix, which was highlighted in our previous work [20], and (b) the active cutting method involves shearing by the tool blade, indicating the dominance of ductile machining. The machined surfaces of the CFRPs (see Figure 10(c1,d1)) are free from naked and/or realigned rCFs, indicating that the PLA matrix likely did not experience overheating during dry milling. The non-existence of such defects could result in a smoother, more uniform finish that enhances the samples’ overall quality and potential performance in practical applications. As addressed by [29], there is a correlation between surface quality and mechanical performance, such that mechanical performance can be improved as the surface quality improves. Despite the absence of delamination, tearing, and layer lines, there are some occurrences of void formation around the rCFs for PLA/5% rCF and PLA/5% rCF/0.5% GNP samples along the tool rotational sweeping direction, but mostly only on the distal side. This is because when the tool blade approaches a region in the PLA matrix with rCFs, the rCFs act as a hard inclusion in the matrix, and thus a void is generated around the fiber (further providing evidence of ductile machining). However, the void from the tool approach side is filled with the softened PLA matrix due to the compressive forces being exerted by the tool on the matrix. On the contrary, while the tool is exiting the fiber site after cutting, the PLA matrix is pulled along the tool sweeping direction, thus leaving a void at the tool distal side around the rCFs being cut. This phenomenon occurs less in the case of the PLA/5% rCFs/0.5% GNPs (Figure 10(d1)) compared to PLA/5% rCFs (Figure 10(c1)) due to enhanced lubrication between the tool and the material being cut. Furthermore, by comparing the surface morphologies of the machined samples without GNPs, as shown in Figure 10(a1,c1), with the GNP-based machined samples in Figure 10(b1,d1), respectively, it can be seen that no chip deposition is observed in the GNP-based samples. Since the chip deposition is mainly governed by the friction between the tool and the workpiece, chips get sandwiched between the tool and the workpiece, leading to chip deposition on the machined surface. Negligible chip deposition in the cases of the GNP-based machined samples further presents the evidence of reduced friction or improved lubrication between the tool and the workpiece materials while machining these composites.

**Figure 11:**
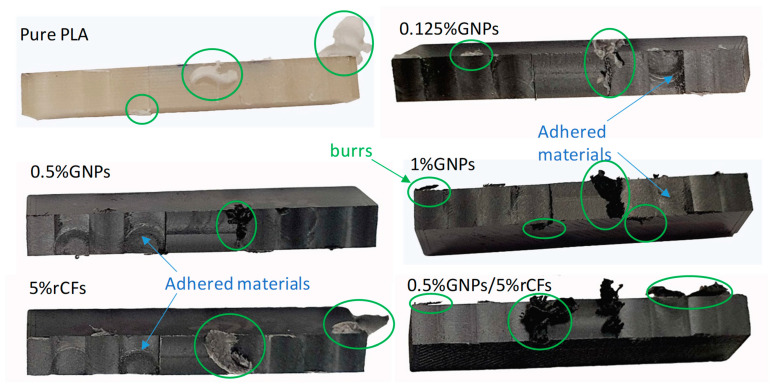
Machined surfaces: burrs.

Despite the improvement in the surface quality of the machined surfaces, the formation of burrs and chips during dry milling can deteriorate the surface quality. This is evident from the presence of the burrs adhering to the machined surfaces, as illustrated in Figure 11. The similar burr formation patterns were observed by [23,24], where the burrs remain intact with the machined edges due to the upsetting phenomenon in which the material being cut is plastically deformed and deposited on the side and exit edges. It can be noticed that the burrs that are left (as circled in green) with the edges generated during the last milling pass can be removed by the next overlapping pass.

## 4. Conclusions

This research investigated the development of composite polymers for MEX printing. PLA reinforced with GNPs and/or rCF composites was mixed through ball mill mixing, and then MEX filaments were developed using an extrusion process. The influence of incorporating GNPs and rCFs into PLA on the mechanical performance and machinability analysis was studied. Tensile testing was conducted to evaluate the mechanical properties of the developed materials. Dry milling post-processing was conducted to study the machinability of the developed materials, with the aim of enhancing the MEX-printed part’s surface roughness. From the results of this study, we can conclude the following:

- SEM images revealed that filaments of pure PLA and PLA/GNPs showed smooth surface topographies.
- Incorporating rCFs into the PLA results in a rougher surface than pure PLA and PLA/GNP filaments.
- The SEM images of the as-built samples from both pure PLA and 0.5 wt.% GNPs showed no voids, cracks, or delamination defects in the intralayer morphology. However, delamination defects between layers (interlayer) were obvious for PLA/0.5% GNP as-built samples.
- As-built samples with rCF incorporation showed voids and naked rCFs on both surfaces and interlayer and intralayer morphology.
- The mechanical properties of the PLA and PLA/GNPs are similar, with GNP loading up to 0.5 wt.%. However, as the GNP loading increased (1 wt.%), the ultimate strength reduced, and composites became more brittle.
- While the stiffness was increased by approximately 15% in the PLA/5% rCF sample compared to the pure PLA sample, the strength and ductility were reduced. The mechanical properties of the PLA/5% rCF sample were unaffected by adding 0.5 wt.% GNPs.
- Post-processing dry milling showed an enhanced surface quality of MEX-printed parts in terms of surface roughness and the absence of deficiencies such as delamination and layer lines. The Sz parameter is more sensitive than the Sa in capturing the surface asperities while machining polymer composites. Up to ~50% reduction in Sz can be achieved during dry machining with the addition of GNPs in the composites. The lack of apparent cavities and grooves indicates that the main machining mechanism was ductile removal.
- It could be claimed that the addition of GNPs could enable the dry machining of the PLA composites with better surfaces than the neat PLA.

## Figures and Tables

**Table 1:** Material characteristics.

Material	Characteristic	Value
PLA	Density	1.24 g/cm^3^
	Melting temperature	145–160 °C
GNPs	Purity, %	99.9%
	density	2.2 g/cm^3^
	size (thickness)	3 nm
	diameter	1.5 µm
	specific surface area	800 m^2^/g
rCFs	purity	97%
	bulk density	0.35 Kg/L
	average length	100 ± 20 µm
	average diameter	7 µm
	tensile strength	3.50 GPa
	tensile modulus	230 GPa

**Table 2:** Weight loadings of the PLA and GNPs.

No.	Material	Weight %, wt.%		
		PLA	GNPs	rCFs
1	PLA	100	-	-
2	PLA/0.125% GNPs	99.875	0.125	-
3	PLA/0.5% GNPs	99.5	0.5	-
4	PLA/1% GNPs	99	1	-
5	PLA/5% rCFs	95	0	5
6	PLA/0.5% GNPs/5% rCFs	94.5	0.5	5

**Table 3:** Ball mill mixing process parameters.

Variable	Setting
Jar type and size	Alumina (1 L)
Mixing balls	ZrO_2_ balls (10 mm)
Speed	320 rpm
Forward mixing time	10 min
Standby time	10 min
Reverse mixing time	10 min
Mixing time	90 min

**Table 4:** Employed parameters for filaments’ extrusion process.

Variable	Setting
Filament diameter	1.75 mm
Temperature of the heating zones	170, 185, 190, and 170 °C
screw speed	6.5 rpm
Cooling, fan speed	85–95%

**Table 5:** MEX process settings.

Variable	Setting
Extrusion temperature	220 °C
Build plate temperature	65 °C
Print velocity	45 mm/s
Layer thickness	0.2 mm
Infill	100%
Raster angle	±45°
Nozzle type	hardened steel nozzle
Nozzle diameter	0.4 mm

**Table 6:** Machining parameters.

Parameter	Setting
tool type	End mill carbide
tool diameter, mm	5
Flute	4
Cutting velocity, m/min	100
Feed rate, mm/tooth	0.02
depth of cut, mm	0.2 mm

**Table 7:** Tensile testing of mechanical properties.

Material	E (GPa)	$Y_{0.2\%}$ (MPa)	UTS (MPa)	$\varepsilon_{b}$ (%)	Weight (g)
PLA [20]	3.25 ± 0.04	42.36 ± 1.32	51.31 ± 1.11	4.42 ± 0.32	9.92 ± 0.02
PLA/0.125% GNPs	3.32 ± 0.04	43.53 ± 0.22	50.34 ± 0.76	4.59 ± 0.55	9.97 ± 0.02
PLA/0.5% GNPs	3.27 ± 0.05	43.98 ± 0.62	50.78 ± 0.57	4.24 ± 0.34	9.94 ± 0.02
PLA/1% GNPs	3.31 ± 0.04	42.05 ± 2.34	47.53 ± 1.38	3.3 ± 0.14	10.04 ± 0.01
PLA/5% rCFs [20]	3.74 ± 0.05	38.01 ± 0.83	45.67 ± 0.44	3.74 ± 0.33	9.93 ± 0.01
PLA/0.5% GNPs/5% rCFs	3.76 ± 0.1	38.82 ± 1.16	45.55 ± 0.59	3.47 ± 0.32	9.97 ± 0.05

## Data Availability

The original contributions presented in this study are included in the article. Further inquiries can be directed to the corresponding author.
